
JOURNAL INFORMATION
==============================
NLM Title Abbreviation: ScientificWorldJournal
Journal ID: ScientificWorldJournal
NLM Journal ID: 101131163
Journal ID: TSWJ

The Scientific World Journal

ISSN: 2356-6140
EISSN: 1537-744X
Publisher: Wiley

ARTICLE INFORMATION
==============================
PMCID: PMC6084246
DOI: 10.1100/tsw.2001.444
Article ID: 905492
Article version: 1
Subjects: Extended Abstract

Gender Differences in the Development of Pulmonary Hypertension (PH)

Vijay Palaniswamy
Tighe Shannon
Jha Deepika
Johnson Tomalyn
Sharp Thomas G.
Packer C. Subah
Indiana University School of Medicine, Indianapolis, IN, USA
Publication date: 2001
Electronic publication date: 2001 Dec 19
Volume: 1
First page: 47
Last page: 47
Copyright: Copyright © 2001 Palaniswamy Vijay et al.
Copyright year: 2001
License: This is an open access article distributed under the Creative Commons Attribution License, which permits unrestricted use, distribution, and reproduction in any medium, provided the original work is properly cited.
License URL: https://creativecommons.org/licenses/by/3.0/

==============================
4th International Meeting on Calcitonin Gene-Related Peptide (CGRP)
*TheScientificWorld* (2001) 1(S1), 47
ISSN 1532-2246; DOI 10.1100/tsw.2001.444

### GENDER DIFFERENCES IN THE DEVELOPMENT OF PULMONARY HYPERTENSION (PH)

Palaniswamy Vijay, Shannon Tighe, Deepika Jha, Tomalyn Johnson, Thomas G. Sharp, and C. Subah Packer
Indiana University School of Medicine, Indianapolis, IN

### INTRODUCTION

PH is a progressive disease that results in right ventricle failure and death. We investigated the levels of a vasodilator, adrenomedullin (ADM), and response to various dilatory agonists during the development of experimentally induced PH in male and female rats.

### METHODS

SD rats of both sexes were injected with 60 mg/kg b wt monocrotaline (MCT) and were terminated after 3 weeks. Plasma ADM (pg/ml) and ET-l (ng/ml) levels were determined. At the maximal contractile responses to NE or ET-1, the rings were exposed to $5 \times 10^{-6}$ M ACh or $9 \times 10^{-9}$ M ADM or $1.3 \times 10^{-8}$ M calcitonin gene related peptide (CGRP).

### RESULTS

MCT at the given dose is highly toxic to male rats when compared to female rats that yielded in high mortality (35% vs. 0.03%, $p = 0.0001$). Vascular endothelial damage caused by MCT triggers 12× more ET-l synthesis/secretion in male rats whereas only 1.3× increase ADM levels. In the case of female rats, ADM secretion is increased with the increase in ET- 1. Relaxation in response to ACh, ADM, and CGRP was endothelium-dependent. All three relaxing agents produced less relaxation of hypertensive pulmonary arterial rings. ADM and CGRP induced relaxation of NE contractions was 2.4× greater in female compared to male control vascular rings ($p < 0.004$) and 5.5× greater in female compared with male hypertensive rings ($p < 0.005$).

### CONCLUSION

We conclude that gender plays an important role in the development of PH and that female rats exhibit a protective effect in the disease progression suggesting that sex hormones may influence the pathophysiology of the PH.
